# Major depression and enhanced molecular senescence abnormalities in young and middle-aged adults

**DOI:** 10.1038/s41398-019-0541-3

**Published:** 2019-08-21

**Authors:** Breno S. Diniz, Charles F. Reynolds III, Etienne Sibille, Mariska Bot, Brenda W. J. H Penninx

**Affiliations:** 1 Adult Neurodevelopment and Geriatric Psychiatry Division, Centre for Addiction and Mental Health, Toronto, ON Canada; 2 Department of Psychiatry, Faculty of Medicine, University of Toronto, Toronto, ON Canada; 3 Campbell Family Mental Health Research Institute, Centre for Addiction and Mental Health, Toronto, ON Canada; 4 Department of Psychiatry, University of Pittsburgh School of Medicine, Pittsburgh, PA USA; 5 Department of Pharmacology and Toxicology, University of Toronto, Toronto, ON Canada; 6 Amsterdam Public Health Research Institute, Department of Psychiatry, Amsterdam UMC, Vrije Universiteit, Amsterdam, The Netherlands

**Keywords:** Depression, Prognostic markers

## Abstract

Recent evidence suggests a significant overlap in biological changes between major depression and aging across the lifespan. We aim to evaluate the impact of a major depressive episode on the Senescence-Associated Secretory Phenotype (SASP) index, a dynamic secretory molecular pattern indicative of cellular senescence. We also tested the potential moderators of the association between major depression and the SASP index. We included 1165 young and middle-aged adults (527 with a current major depressive episode (cMDE) and 638 with no lifetime history of depression) from a community-based cohort from the Netherlands. We calculated the SASP index based on a previously developed composite index involving 19 biomarkers. cMDE had higher SASP index values (t_(1163)_ = 2.93, *p* = 0.003) compared to controls in the univariate analysis. After controlling for sociodemographic and somatic health covariates, there was no significant association between cMDE and SASP index (F_(1,1158)_ = 1.09, *p* = 0.29). Those with the most severe depressive episodes had significantly higher SASP indices compared to those with mild-to-moderate cMDE and controls (F_(2,1162)_ = 6.73, *p* = 0.001). We found a significant interaction between cMDE and overweight (F_(1,1164)_ = 5.1, *p* = 0.028): those with comorbid cMDE and overweight had the highest SASP index. Our study demonstrated a complex interaction between cMDE and medical morbidity, especially overweight, on the SASP index, suggesting that their coexistence aggravate age-related biological processes. Moreover, higher SASP index can be a biomarker for more severe depressive episodes.

## Introduction

Major depressive disorders (MDD) across the lifespan are among the most common mental disorders in the general population, with a lifetime prevalence ranging from 5 to 15%^1,2^. Aside from its high prevalence, MDD is the second most disabling health condition worldwide (after cardiovascular disease), has a chronic and relapsing course, poor treatment response and is linked to higher risk of developing chronic and disabling conditions^3^. For example, clinical and epidemiological studies showed that subjects with MDD are at increased risk for cardiovascular and metabolic diseases (e.g., obesity, diabetes hypertension, myocardial infarction, and metabolic syndrome), cerebrovascular diseases, cognitive impairment, and development of Alzheimer’s disease (AD) and Vascular dementia (VaD)^4–6^. MDD is also linked to increased risk of frailty, functional impairments, and premature mortality^7,8^. These negative health outcomes are commonly observed during the non-pathological aging process, suggesting that subjects with MDD present with, or are at higher risk for, an “enhanced clinical aging phenotype”^9^.

The molecular mechanisms linking MDD to poorer health outcomes are not well understood, but probably involve the interaction of depression with medical morbidity, and age- or senescence-related biological processes^10,11^. Senescence is a complex process that involves various systemic cellular and tissue changes that culminate with reduced reserve, regenerative ability, and capacity to antagonize toxic insults^12^. Although senescence is a non-pathological process, the build-up and accumulation of senescence-related changes in an organism have been associated with metabolic dysfunction, changes in the fat distribution, increased frailty, cognitive decline, and sarcopenia^13–16^. Therefore, understanding how MDD and somatic health variables affect biological mechanisms associated with senescence can help explain why these conditions increase the risk of age-related disorders and disability, as well as identifying novel targets for intervention.

Changes in the cellular secretome pattern are one of the hallmarks of senescence and commonly referred to as Senescence-Associated Secretory Phenotype (SASP)^17,18^. The SASP comprises multiple signaling proteins involved in the immune-inflammatory response, cell growth, control of cell cycle and apoptosis, cell-to-cell communication, and tissue remodeling^19^. SASP changes are dynamic and can occur in response to activated oncogenes, metabolic insults, biological and psychosocial stress, and damage/danger signals. The enhanced secretion of SASP signaling proteins after a deleterious stimuli have been previously associated with the development of canonical hallmarks of cellular aging (e.g., telomere attrition, higher expression of p16^ink4^); likewise, cellular senescence changes can drive the cellular secretome towards an enhanced SASP profile^20–23^. Under non-pathological circumstances, protective cellular responses are rapidly activated to restore the cellular homeostasis^24^. Nonetheless, the persistence of deleterious stimuli can lead to the chronic activation of senescence response, leading to a positive feed-forward loop with enhanced SASP and other pro-senescence cellular changes. Thus, SASP can be viewed as a dynamic molecular pattern that reflects the current level of cellular senescence of the organism^25^.

Building upon past proteomic studies from our group on peripheral blood samples, we developed the SASP index^26–28^. The SASP index is composed of 22 independent circulating proteins that are part of the secretome of aging cells and that were previously described as a cellular secretory pattern common to different senescent stimuli^17,29^. The SASP index can be viewed as a measure of background cellular senescence with higher SASP index reflecting greater senescence. In our initial study, we found that older adults with major depression had significantly higher SASP index compared to never- depressed, healthy controls^27^. Also, the SASP index was positively correlated with higher age and greater medical comorbidity burden. However, that study had important limitations, since it included a small sample size and was restricted to older adults recruited at a specialized center for the treatment of late-life depression. Thus, our findings might not generalize to the general population, nor could we evaluate whether SASP index changes were already present in younger adults and test whether other clinical variables contributed or even moderated the relationship between SASP and depressive symptoms.

In the current study, our primary goal was to evaluate the association between the SASP index and major depression in a large, population-based cohort of young and middle-aged adults. Our primary hypothesis was that subjects with a current MDD diagnosis would have a higher SASP index compared to never-depressed subjects, indicating greater cellular senescence. We also explored the association between the SASP index and characteristics related to the depressive episode (e.g., symptom severity and depression subtype), and whether demographic and somatic health variables contributed to the association between MDD and cellular senescence.

## Methods

### Study participants

Data were derived from the Netherlands Study of Depression and Anxiety (NESDA), an ongoing longitudinal cohort study on the predictors, course, and consequences of depressive and anxiety disorders. The NESDA sample consists of 2981 participants aged 18–65 years, comprising persons with and without depressive and anxiety disorders. Participants were recruited from the general population (*n* = 564), primary care (*n* = 1610), and specialized mental-health care (*n* = 807). Between September 2004 and February 2007, all participants visited one of the research centers to complete the 4-h baseline assessment, which included a face-to-face interview, written questionnaires, and biological measurements. A detailed description of the NESDA study design can be found elsewhere^30^. In this analysis, we use a subset of 1165 subjects who had SASP biomarkers measured in serum samples, and a current diagnosis of a major depressive episode (cMDE, *n* = 527) or no history of major depression (Controls, *n* = 638).

The research protocol was approved by the Ethical Committee of the participating centers, and all participants provided written informed consent.

### Depressive symptoms assessment

The presence of depressive symptoms was assessed in all participants using the self-reported 30-item Inventory of Depressive Symptomatology (IDS^31^,). Besides the assessment with the IDS, all participants also completed the Composite Interview Diagnostic Instrument (CIDI, version 2.1^32^,) by trained research staff. The CIDI is a validated diagnostic interview for mental disorders based on the DSM-IV. In the current study, we defined current major depressive episode (cMDE) as subjects with the CIDI diagnostic criteria of a major depressive episode (based on DSM-IV criteria). Those with no lifetime history of a major depressive episode were included as a comparison group in the analyses.

### Additional mental-health variables

The presence of anxiety symptoms was assessed by the 21-item Beck Anxiety Inventory (BAI)^33^. History of childhood trauma was evaluated by the Childhood Trauma Interview, rating on a 0–8 score the presence of emotional neglect, psychological abuse, physical abuse, and sexual abuse before the age of 16 years^34^. History of alcohol dependence or abuse was based on the DSM-IV criteria as ascertained through CIDI administration.

### Sociodemographic and somatic health variables

We included age, sex, self-reported ancestry, smoking status based on the pack-year measure, body mass index (BMI), physical activity, count of the number of chronic diseases for which subjects received medical attention in the last year, ankle/brachial index (an indirect measure of peripheral artery disease), systolic and diastolic blood pressure (summarized over two measurements in mmHg) as sociodemographic and somatic health variables. Weight and height were measured by trained staff to calculate BMI. Physical activity was assessed with the International Physical Activity Questionnaire and expressed in 1000 metabolic equivalent minutes per week^35^.

### SASP index biomarkers

As described in our previous study^27^, the Senescence-Associated Secretory Phenotype Index (SASP Index) is composed of 22 independent circulating proteins that are part of the secretome of aging cells and that were previously described as a cellular secretory pattern related to different senescent stimuli^17^. Greater SASP index reflects greater molecular senescence values. The list of SASP index biomarker is IGFBP6, IGFBP2, CCL4, IL-1β, GM-CSF, PLGF, Angiogenin, MIF-1, MIP-1A, Gro-α, IL-6, MCP-4, Gp130, ICAM-1, MCP-1, IL-8, MIP-3A, Osteoprotegerin, TIMP-1, uPAr, TNFRI, and TNFRII.

The serum biomarkers were analyzed using a multiplex panel of 243 markers (Myriad RBM DiscoveryMAP 250+), including the 22 biomarkers forming the SASP index. The laboratory analyses were done at the MyriadRBM facility (Austin, TX, USA). The biomarkers were measured by LUMINEX technology. This method measures analytes using a flow cytometric system. The process was fully automated (for white paper http://rbm.myriad.com/scientific-literature/white-papers/quality-control-white-paper/). Each batch also contained three duplicate control samples with different protein concentrations, giving an average inter- and intra-assay variability of 10.6% (range 5.5–32.5%) and 5.6% (range 2.5–15.8%), respectively. All laboratory analysis was done in duplicate. A detailed description of the biomarkers analysis can be found elsewhere^36^.

From the 22 biomarkers included in the SASP index, three biomarkers (GM-CSF, Il-1β, and PLGF) had more than 30% of missing values and were not included in the SASP index. IL-6 also had more than 30% of missing data in the multiplex panel. However, IL-6 levels were also analyzed by ELISA methodology yielding much less missing data (<0.5) and were included in the SASP index. Multiple imputation models were used to input missing data values. Therefore, a total of 19 biomarkers were used to calculate the SASP index.

The raw biomarker data were log2 transformed and standardized to the z-score. We calculated the SASP index for each participant based on the following regression formula:

$${\boldsymbol{SASP}} = {\boldsymbol{\beta }}_1{\boldsymbol{x}}_1 + \ldots + {\boldsymbol{\beta }}_{22}{\boldsymbol{x}}_{22} + {\boldsymbol{\varepsilon }}$$

Where β_x_ is the individual weight and x_x_ is the standardized value of each biomarker included in the SASP index. The biomarker weight for each biomarker was based on the first component of a principal component analysis and derived from our previously published study^27^. The SASP index mean was centered at 0, with a standard deviation of 1 in the whole sample. Supplementary table 1 shows the biomarkers included in the SASP index, their respective weights to calculate the SASP index, and their mean values in this sample.

### Statistical analysis

Prior to statistical analysis, we evaluated the distribution of data and they followed parametric distribution. We carried out Student *t*-test or Chi-square analyses to evaluate the effect of cMDE episode on SASP index, sociodemographic, mental and somatic health variables. We next evaluated if sociodemographic, mental and somatic health variables that were associated with cMDE contribute to the association between MDD and SASP index using a generalized linear model. We further carried out Pearson analyses to evaluate the strength of the correlation between the SASP index and sociodemographic, mental and somatic health variables. Finally, we tested the association between specific characteristics of the depressive episode (e.g., psychopathological characteristics, recurrence of the depressive episode, and severity of the current depressive episode) and SASP index values. We further tested whether demographic and somatic health variables moderated the effect of major depressive episode and SASP index values. All analyses were done with the Statistical Package for Social Science (SPSS v24, Chicago, USA).

## Results

### Current MDE and SASP index

The sociodemographic and clinical characteristics of the sample are reported in Table 1. Among subjects with cMDE, 21.4% (*n* = 113) reported taking antidepressant medication. Antidepressant use did not significantly influence the SASP index among individuals with MDD (t = 0.016, df = 525, *p* = 0.98); thus, all individuals in the cMDE group were included in the analysis. As expected, the individuals with a cMDE had significantly higher SASP index compared to the control group (t_(1163)_ = 2.93, *p* = 0.003, Cohen’s d = 0.18).

**Table 1:** Associations between MDD presence and SASP index, sociodemographic, and clinical variables

	Current MDD (*N* = 527)	Controls (*N* = 638)	Statistics	*p*-value
	Mean ± SD	Mean ± SD		
SASP index	0.10 ± 1.04	−0.07 ± 0.96	t_(1163)_ = 2.93	0.003^a^
Chronological age (years)	42.3 ± 12.4	39.9 ± 14.5	t_(1163)_ = 2.98	0.002
Sex				
Male	178	234	X^2^_(1)_ = 1.05	0.32
Female	349	404		
North-European ancestry				
No	29	21	X^2^_(1)_ = 3.43	0.08
Yes	498	617		
Inventory of Depressive Symptoms—30 (IDS-30)	34.7 ± 11.2	11.5 ± 9.3	t_(1163)_ = 38.68	<0.001
Childhood trauma (NEMESIS)	1.2 ± 1.3	0.5 ± 0.9	t_(1163)_ = 12.56	<0.001
Beck Anxiety Inventory (BAI)	18.8 ± 10.9	6.5 ± 7.3	t_(1163)_ = 23.05	<0.001
Alcohol use disorder (DSM-IV)				
No	363	525	X^2^_(1)_ = 28.62	<0.001
Yes	164	113		
Tobacco smoking (pack/year)	12.7 ± 17.7	7.1 ± 13.2	t_(1163)_ = 6.21	<0.001
Mean systolic blood pressure	137.0 ± 20.2	136.2 ± 19.9	t_(1163)_ = 0.66	0.51
Mean diastolic blood pressure	82.5 ± 11.3	80.2 ± 11.3	t_(1163)_ = 3.54	<0.001
Ankle/Brachial index	1.1 ± 0.2	1.1 ± 0.1	t_(1163)_ = 0.79	0.42
Body Mass Index (BMI)	26.4 ± 5.7	25.1 ± 4.8	t_(1163)_ = 4.31	<0.001
Number of chronic diseases	1.1 ± 1.2	0.7 ± 0.9	t_(1163)_ = 7.07	<0.001
Physical activity (total MET/minutes a week)	3,401.8 ± 3,041.6	3,950.5 ± 3,142.3	t_(1163)_ = 2.68	0.004

^a^After adjustment for potential confounding variables (age, childhood trauma, BAI scores, alcohol use disorder, tobacco smoking, diastolic blood pressure, number of chronic diseases, physical activity), there was no significant differences in the SASP index values between cMDE and controls (F_(1,1158)_ = 1.09, *p* = 0.29)

The association between cMDE and SASP can be influenced by different variables that are associated with this condition. We carried out a generalized linear model incorporating the demographic, somatic, and other mental-health variables that were significantly associated with cMDE in the univariate analysis (Table 1). The association between cMDE and SASP index was not statistically significant after controlling for potential confounding variables (F_(1,1158)_ = 1.09, *p* = 0.29). In the multivariate model, BMI, age, number of chronic diseases, cigarette pack-years were the variables that had the most significant influence on the SASP index.

### The effect of cMDE subtype, recurrence of the depressive episode, and severity of depression on SASP index

Previous analyses of the NESDA cohort have shown that the subtype of the depressive episode (i.e., atypical or melancholic depression) can have a significant impact on somatic health parameters and peripheral biomarkers. For example, melancholic depression was associated with greater HPA-axis dysfunction, while atypical depression was associated with a pro-inflammatory status and poorer metabolic status^37,38^. Therefore, we tested whether the cMDE subtype could also be associated with changes in the SASP index. Based on previous analysis using latent class analysis to define depressive subtypes, 113 individuals were classified as having atypical depression, 87 with melancholic depression, and 327 of individuals with cMDE as having neither an atypical or melancholic episode^37^. Univariate analysis of variance showed an omnibus statistically significant difference of SASP index values between controls and specific subtypes of depression (F_(3,1161)_ = 3.05, *p* = 0.0028), with controls having the lowest SASP index values. Post-hoc analysis did not show significant difference in the SASP index values according to the depressive subtypes (Table 2).

**Table 2:** Clinical characteristics of the depressive episode and SASP index values

Subtype of depressive episode vs. SASP index				
Subtype of depression	*N*	Mean ± SD	Omnibus statistics	
Control	638	−0.07 ± 0.96	F_(3, 1161)_ = 3.05	*p* = 0.028
MDE atypical^a^	113	0.05 ± 0.99		
MDE melancholic^a^	87	0.07 ± 1.13		
MDE not Classified^a^	327	0.12 ± 1.04		

Recurrence of depressive episode vs. SASP index				
	*N*	Mean ± SD	Omnibus statistics	
Control	638	−0.07 ± 0.96	F_(2,1162)_ = 4.77	*p* = 0.009
MDE first episode^a^	247	0.15 ± 1.04		
MDE recurrent episode^a^	267	0.06 ± 1.05		

Severity of depressive episode vs. SASP index				
	*N*	Mean ± SD	Omnibus statistics	
Control	638	−0.07 ± 0.03	F_(2,1162)_ = 6.73	*p* = 0.001
MDE mild/moderate^b^	330	0.006 ± 0.05		
MDE severe/very severe^b^	197	0.24 ± 0.07		

^a^Post-hoc analysis showed no statistically significant difference between the depression subtypes or depressive episode recurrence on the SASP index values

^b^Post-hoc analysis: severe/very severe vs. control (*p* < 0.001); severe/very severe vs. mild/moderate (*p* = 0.028); mild/moderate vs. controls (*p* = 0.6)

We also evaluated the association between major depressive episode recurrence, episode severity and SASP index values. Univariate analysis of variance showed an omnibus statistically significant difference of SASP index, with controls having lower SASP index values compared to those with a single and recurrent MDE (F_(2,1162)_ = 4.77, *p* = 0.009). There was no statistically significant difference between single vs. recurrent major depressive episode in the post-hoc analysis. Finally, we divided the sample according to the severity of the depressive episode based on the IDS-30 scores. Those with a cMDE and scores between 14 and 38 were classified as mild-to-moderate episode; those with scores between 39 and 84 were classified as severe and very severe episode (www.ids-qids.org). Univariate analysis of variance showed an omnibus statistically significant difference of SASP index between groups, with the control group having the lowest SASP index values compared to those with mild-to-moderate and severe and very severe depressive symptoms (F_(2,1162)_ = 6.73, *p* = 0.001). Post-hoc comparison showed that individuals with severe and very severe MDE episode had significantly higher SASP index scores compared to controls (*p* < 0.001) and compared with those having mild-to-moderate cMDE in the post-hoc (*p* = 0.02) (Table 2).

### The effect of demographic and somatic health variables on SASP index

As demographic and somatic health variables were significant covariates in the association between MDD and SASP index, we sought to evaluate the independent association of these variables with SASP index. We focused on the variables measuring chronological age, body mass index, tobacco smoking, diastolic blood pressure, and number of chronic diseases since they were significant covariates in the multivariate model testing for the effect of cMDE on SASP index. Linear regression analysis showed that all these variables, except diastolic blood pressure, were independently associated with SASP, having a small to moderate association with the SASP index (supplementary table 2). Body mass index and age had the strongest univariate correlation with SASP index (BMI: r = 0.37, *p* < 0.001; age: r = 0.32, *p* < 0.001).

Since BMI and chronological age were the variables that had the largest independent impact on SASP index and they have a significant influence on mechanisms related to major depression, we evaluated the interaction of cMDE, BMI, and age on the SASP index value. We first dichotomized the individuals based on BMI (BMI < 24.99, normal weight; BMI > 25, overweight/obesity) and age (18–44 years old, young adults;>45 years old, middle-aged adults). We carried out a general linear model analysis with diagnosis (cMDE vs. control), age or BMI, and the interaction between depression diagnosis*BMI or depression diagnosis*age.

**Fig. 1:**
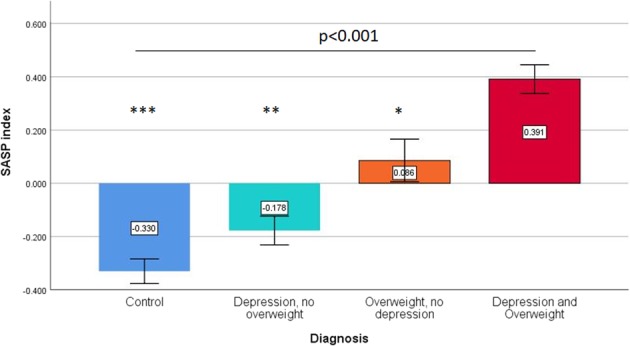
Effect of the depression and overweight on SASP index values. Subjects with current major depressive episode and overweight have the highest SASP index values compared to the other groups (omnibus ANOVA *p*-value < 0.001). We also found a significant interaction between the diagnosis of major depressive episode and weight (*p* = 0.024, see Table 3) on SASP index values. Pairwise comparisons (Depression and Overweight group as reference): *p = 0.001; **p < 0.001; ***p < 0.001

We found a statistically significant interaction between depression diagnosis and BMI (F_(1,1164)_ = 5.1, *p* = 0.028). The individuals in the group cMDE+ overweight/obesity had the highest SASP index in this analysis (Table 3 and Fig. 1). There was no significant interaction between depression diagnosis and age in these individuals (F_(1,1164)_ = 0.17, *p* = 0.6) (Table 3).

**Table 3:** Moderating effect of weight and chronological age on the association between cMDE and SASP index

Chronological age	
cMDE	F_(1,1164)_ = 8.71, *p* = 0.003
Chronological age^a^	F_(1,1164)_ = 107.4, *p* < 0.001
cMDE*Chronological age^a^	F_(1,1164)_ = 0.177, *p* = 0.67

Body mass index	
cMDE	F_(1,1164)_ = 8.71, *p* = 0.003
BMI^b^	F_(1,1164)_ = 96.1, *p* < 0.001
cMDE*BMI^a^	F_(1,1164)_ = 5.1, *p* = 0.024

^a^Chronological age was dichotomized as young (18–44 years old) and middle-age adults (45–65 years old)

^b^BMI was dichotomized as normal weight (BMI < 24.99) and overweight and obesity (BMI > 25)

To further explore the association between depression, overweight and SASP index, we did additional secondary pairwise analyses. The “Depression and Overweight” group had significantly higher BMI scores than the “No Depression, Overweight” group (BMI, 30.34 ± 4.73 vs. 29.01 ± 3.78, t = 3.34, df = 553, *p* = 0.001). In an analysis of covariance, the “Depression and Overweight” group had significantly higher SASP index scores compared to the “No Depression, Overweight” group, after controlling for BMI (F = 25.34, d.f. = 1553, *p* = 0.001). Likewise, the “Depression and Overweight” group had significantly higher IDS scores than the “Depression, No Overweight” group (IDS, 33.01 ± 11.7 vs. 30.34 ± 10.6, t = 2.65, d.f. = 718, *p* = 0.002). In an analysis of covariance, the “Depression and Overweight” group had significantly higher SASP index scores compared to the “Depression, No Overweight” group, after controlling for IDS scores (F = 60.5, d.f. = 1717, *p* < 0.001).

## Discussion

In this study, we aimed to investigate the association between major depression and molecular senescence abnormalities, measured by the SASP index, in a large community-based cohort of young and middle-aged adults. The SASP index was higher (indicating a greater cellular senescence) in the group with a current major depressive episode, although this association was moderated by demographic (e.g., age and sex) and somatic health variables (e.g., overweight, blood pressure, number of chronic diseases, and tobacco smoking). Nonetheless, the SASP index was significantly higher in specific groups of subjects with cMDE, like those with severe depressive episode or those with comorbid overweight and obesity. These findings further suggest that depression and somatic health variables interact and lead to greater cellular senescence abnormalities in young and middle-aged adults and making them more vulnerable to negative health outcomes.

In the multivariate model, several biological and somatic health variables independently predicted SASP index values. Higher BMI, older age, smoking, multi-morbidity, and high diastolic blood pressure are all well-established markers of a worse health condition and major risk factors for general health deterioration and premature death. The strength of the association between these variables and the SASP index were of small to moderate effect size. This is expected since the biology of aging is a complex phenomenon that involves the effect of several factors on multiple biological pathways^18^.

Major depression, metabolic disorders, and cardiovascular disorders have a well-documented clinical and epidemiological association. Overweight significantly increase the risk of a major depressive episode, and vice-versa^5,39–42^. All these conditions have been associated with greater pro-inflammatory status, higher oxidative stress markers, among other markers of cellular senescence^43–45^. There is a large body of evidence indicating that the adipose tissue is a key mediators of these biological changes and it acts as an endocrine organ regulating metabolism and inflammatory response locally and systemically^46,47^. Adipose tissue actively secretes pro and anti-inflammatory cytokines and plays a major role in the control of the insulin signaling cascade, insulin resistance, and metabolic control. Many of the cytokines and insulin signaling cascade proteins that are secreted by adipocytes also play a major role in the SASP. Obesity is associated with changes in the secretome of adipocytes, leading to greater production of pro-inflammatory cytokines that can contribute to increased SASP index in obese subjects^42,48^. Other biological abnormalities linked to overweight and obesity, including insulin resistance, mitochondrial dysfunction, and an increase in oxidative status, are also common during a major depressive episode^49–52^. We also showed that the effect of overweight and depression on SASP index was independent of the BMI or depressive symptoms in the secondary analysis. Altogether, our findings suggest that the interaction of depression and overweight can significantly potentiate senescence abnormalities in young and middle-aged adults, as measured by the SASP index. It can also inform a mechanistic framework of why individuals with comorbid obesity and depression have a greater disability and worse long-term health outcomes in the general population.

Our findings can also have potential therapeutic implications. For example, metformin is a drug that is commonly used for the treatment of type 2 diabetes and can have anti-senescence effects by modulating key intracellular pathways related to SASP (e.g., NFκB and p53) and reducing circulating SASP^53–55^. Metformin can also lead to weight loss in overweight and obese diabetic and non-diabetic patients^56^. Although it does not have an antidepressant effect, it can be used as adjuvant therapy in overweight individuals with major depression to reduce circulating SASP proteins, improve molecular senescence parameters, and improve the antidepressant response in this specific group of individuals. Also, the use of senolytics can help to mitigate many of the negative health outcomes related to major depression, including the development of several age-associated medical disorders (e.g., cardiovascular disease) or neuropsychiatric disorders (e.g., Alzheimer’s disease). Although speculative at this moment, future studies need to be designed to address these hypothesis. A better understanding of senescence-related changes and the dynamics of SASP index in major depression can have a significant translational and practical impact since they can be new therapeutic target for drug repurposing and to monitor the therapeutic effect of drugs in subgroups of patients with major depression.

Tobacco smoking has multiple biological effects including the induction of a chronic pro-inflammatory status, increased oxidative stress, telomere shortening, DNA damaging effects, and other cellular oncogenic effects^57–59^. These are pro-aging effects that can contribute to changes in SASP regulation and accelerate senescence changes. Higher medical multi-morbidity is a marker of poorer health, is more common at older ages, and associated with increased pro-inflammatory status^60^. Our findings suggest that enhanced senescence is a common biological feature or a consequence of several different conditions that converge towards worse health parameters or outcomes. Within this context, the SASP index can be regarded as a molecular indicator of background senescence or general health status in one individual. Moreover, the SASP index can be useful to identify those who are more vulnerable to age-related negative health outcomes in clinical practice.

The search for specific biological mechanisms underlying different presentations of the depressive disorder is an area of intense investigation. For example, previous studies suggest that depressive episode with melancholic features had more intense HPA dysfunction, while those with an atypical episode have more intense inflammatory and metabolic changes^37,38,61,62^. Despite their importance, these results remain elusive and not commonly replicated in different cohorts. In the current analysis, characteristics of the depressive episode like recurrence or subtype (melancholic or atypical) had no significant impact on SASP index values. In contrast, we found that the SASP index was significantly higher in those with a more severe depressive episode. Our findings suggest that greater cellular senescence may be an unspecific marker of episode severity, independent of recurrence of depression or depressive psychopathological presentations.

In our previous study restricted to older subjects, we found a significant increase in the SASP index in older adults with major depression, independent of clinical, and other biological variables^27^. We further showed that the SASP index was significantly associated with cognitive impairment, in particular, executive dysfunction and attentional deficits. However, we were not able to fully replicate our initial findings in the current study since the association between the SASP index, and depressive symptoms were not statistically significant after adjustment for clinical and biological variables. The distinct results between studies have important implications. First, as the age groups between studies do not overlap (young and middle-aged vs. older adults), these results suggest that major depression probably has distinct mechanisms (or biomarker-related changes) along the lifespan, consistent with the concept that the pathophysiology of major depressive disorder is highly heterogeneous. Importantly, we found a significant effect of chronological age on SASP index, with middle-aged individuals having a significantly higher SASP index values compared younger adults. There is a complex interaction between age, depression, medical comorbidities (e.g., obesity, high blood pressure, number of chronic medical diseases) that can create a feedback loop that perpetuates the abnormal regulation of SASP and amplifies senescence responses at older ages. We, thus, suggest that the changes in SASP may be greater and significant in older than younger adults, reflecting that accumulation of systemic, pro-senescence stimuli, and be a surrogate molecular marker for poorer systemic (e.g., overweight, hypertension, smoking) and brain health (e.g., cognitive impairment and structural brain changes).

The SASP proteins were measured in the serum, and their exact cellular or tissue sources are unknown. SASP proteins can be secreted by different cells (e.g., leukocytes, adipocytes, muscle cells, pancreatic β-cells, and glia) under physiologic and pathologic conditions. Despite the lack of cellular source specificity of SASP protein, after their secretion into the circulation, they act in concert leading to senescence changes at local and distant sites, increasing the vulnerability of tissues to additional damage and senescence changes. Therefore, the SASP index can be viewed as a dynamic measure of cellular senescence status of an individual. The SASP index is composed by several inflammatory cytokines and it can be viewed as biased towards measuring inflammatory processes and not senescence. However, it is worth noting that although low-grade, sterile inflammation is a key aspect of senescence^63^, it is one among many others biological abnormalities related to senescence (e.g., disruption in metabolic control, decreased insulin sensitivity, impaired tissue remodelling, cell growth, cell cycle control, and arrest). Molecules related to these senescence mechanisms (e.g., IGFBP-2 and 6, angiogenin, GROa, TIMP-1, placental growth factor) are included in the SASP index. Thus, the biological processes of covered by the SASP index is broader than only inflammation and in line with the complexity of senescence.

There are also other limitations of this study. We were not able to include all biomarkers from the original SASP index, due to the large frequency of measures below the assay detection limit. The absence of these markers (IL-1β, GM-CSF, and PLGF) could have significantly influenced the calculation of the SASP index for each subject and consequently may have affected the current results. Also, as the blood biomarkers were only available for baseline assessment, we were not able to test changes in the SASP index over time. Nonetheless, the large sample size, recruited from the general population, with a wide age range, and the careful phenotypic characterization of this study sample are major strengths of our study.

Senescence is a universal, non-pathological phenomenon. However, the accumulation of senescence changes can lead or predispose to chronic diseases and worse health outcomes. The understanding of how age-related biological changes interact at a molecular level with demographic, clinical, and biological variables could provide a novel conceptual framework for the risk of developing chronic diseases (including cardiovascular, endocrine-metabolic, cancer, and neurodegenerative disorders) with aging. Also, it can help explaining why different medical and psychiatric conditions, with very different basic pathophysiologic mechanisms, can significantly affect the underlying aging process and increase the risk of disability, negative health outcomes, and premature mortality in the general population. Finally, with the emergence of novel therapeutic compounds targeting senescence (e.g., senolytic drugs), our findings can guide the development of novel therapeutic interventions to mitigate the risk of negative outcomes associated with different medical conditions^64,65^. Likewise, the SASP index can be a useful surrogate biomarker to evaluate the efficacy of these interventions in different pathologic conditions.

## Supplementary information

Supplementary Information.
