# Supplementary material for: Major depression and enhanced molecular senescence abnormalities in young and middle-aged adults
Source: Transl Psychiatry. 2019 Aug 21;9:198. doi: 10.1038/s41398-019-0541-3 (PMC6704136; doi:10.1038/s41398-019-0541-3)
Supplement: Supplementary file 1 — Supplementary Information. [file 41398_2019_541_MOESM1_ESM.docx]

Supplementary table 1 – The SASP index biomarkers and their respective weights.

| SASP index biomarkers | Weight |
| --- | --- |
| Angiogenin | -0.268 |
| CCL4 | -0.431 |
| Gp130 | 0.152 |
| Gro-α | -0.050 |
| ICAM-1 | 0.265 |
| IGFBP2 | 0.199 |
| IGFBP6 | -0.598 |
| IL-6 | -0.015 |
| IL-8 | 0.324 |
| MCP-4 | -0.003 |
| MIF-1 | -0.260 |
| MIP-1A | -0.050 |
| MIP-3A | 0.373 |
| Osteoptogerin | 0.453 |
| TIMP-1 | 0.512 |
| uPAr | 0.527 |
| TNFRI | 0.557 |
| TNFRII | 0.591 |

Supplementary table 2: The effect of sociodemographic and somatic health variables on SASP index in the multivariate linear analysis.

|  |  | | t | Sig. | | 95.0% Confidence Interval for B | | |
| --- | --- | --- | --- | --- | --- | --- | --- | --- |
|  | B | Std. Error |  |  |  | Lower Bound | | Upper Bound |
| (Constant) | -2.113 | .207 | -10.230 | | <0.000 | | -2.518 | -1.708 |
| Age | .011 | .002 | 4.766 | | <0.000 | | 0.006 | 0.015 |
| Diastolic blood pressure (mmHg) | .002 | .003 | .907 | | 0.364 | | -0.003 | 0.008 |
| Body Mass Index (BMI) | .050 | .005 | 9.181 | | <0.000 | | 0.039 | 0.061 |
| number of chronic diseases | .125 | .026 | 4.718 | | <0.000 | | 0.073 | 0.176 |
| Tobacco smoking (pack/years) | .010 | .002 | 5.285 | | <0.000 | | 0.006 | 0.013 |
| Adjsuted R^2^=22.1% | | | | | | | | |
